# MicroRNAs in Obesity, Insulin Resistance, and Type 2 Diabetes: Mechanistic Insights and Translational Perspectives

**DOI:** 10.3390/ijms27146501

**Published:** 2026-07-22

**Authors:** Tamires M. Zanotto, Mario J. A. Saad

**Affiliations:** 1 Department of Internal Medicine, State University of Campinas (UNICAMP), Campinas 13083-887, SP, Brazil; tamimz@unicamp.br; 2 Department of Medical Clinics, Obesity and Comorbidities Research Center (O.C.R.C.), State University of Campinas (UNICAMP), Campinas 13083-864, SP, Brazil

**Keywords:** microRNAs, obesity, insulin resistance, type 2 diabetes mellitus, extracellular vesicles, biomarkers, precision medicine

## Abstract

Obesity and type 2 diabetes mellitus (T2DM) are multifactorial disorders characterized by insulin resistance, chronic low-grade inflammation, adipose tissue dysfunction, and multi-organ metabolic impairment. MicroRNAs (miRNAs) act as post-transcriptional gene regulators and play critical roles in metabolic homeostasis, the modulation of insulin signaling, adipogenesis, inflammatory pathways, and energy balance in key insulin-target tissues, including liver, skeletal muscle, and adipose tissue. This review summarizes mechanistic and translational insights into miRNA regulation in obesity, insulin resistance, and T2DM, integrating data from human studies and experimental models on miRNA sequence codes and extracellular vesicle sorting pathways. We focus on the tissue-specific and systemic roles of miRNAs, highlighting their contribution to inter-organ communication and metabolic network regulation. In addition, we emphasize their emerging roles as predictive biomarkers, modulators of treatment response, and promising targets for RNA-based interventions. Advances in sequence-specific miRNA sorting and extracellular vesicle-mediated delivery may provide avenues for therapeutic intervention. However, challenges related to delivery efficiency, tissue specificity, off-target effects, and variability in miRNA quantification remain important barriers to clinical translation. Addressing these limitations may help define the clinical utility of miRNAs as biomarkers and therapeutic targets in metabolic disorders.

## 1. Introduction

Obesity is a multifactorial chronic disease and a major global public health challenge, with rapidly increasing prevalence and significant impact on morbidity and mortality. According to the World Health Organization (WHO), obesity is defined as an excessive accumulation of body fat that increases the risk of multiple comorbidities, particularly in relation to cardiometabolic diseases [1]. It is a central component of metabolic syndrome, a set of interrelated conditions that include insulin resistance (IR), type 2 diabetes mellitus (T2DM), dyslipidemia, hypertension, and central adiposity [2].

Recent global estimates indicate that more than a billion people are currently living with obesity, including approximately 880 million adults and 159 million children and adolescents. In addition, around 35–37 million children under the age of 5 are deemed to be overweight or obese. Prevalence has more than doubled in adults and quadrupled among younger populations since 1990, highlighting the rapid escalation of this condition. Projections suggest a substantial impact in the future, with the number of adults living with obesity expected to exceed 1.5 billion globally by 2035, reflecting a rising trend [1,3].

Obesity is closely associated with IR and the development of T2DM, as the increase in adiposity initiates biological processes that directly disrupt normal insulin action and glucose homeostasis. This process is characterized by chronic low-grade inflammation and adipose tissue dysfunction, leading to the increased secretion of pro-inflammatory cytokines and impaired insulin signaling in key insulin-target tissues, such as the liver, skeletal muscle, and adipose tissue [4,5,6].

Although genetic and environmental factors contribute substantially to disease onset and progression, they do not fully account for the clinical and molecular heterogeneity observed in obesity. This gap has driven growing interest in epigenetic modifications (such as DNA methylation and histone modifications) and post-transcriptional regulatory mechanisms, particularly miRNAs, as critical modulators of the pathophysiology of obesity, IR, and T2DM [7,8,9].

miRNAs are small non-coding RNAs that can regulate gene expression at the post-transcriptional level through binding to complementary messenger RNAs (mRNAs), leading to their degradation or translational repression [7]. Through this mechanism, miRNAs influence key metabolic processes, including insulin signaling, adipogenesis, inflammatory pathways, and energy homeostasis [8]. In obesity and T2DM, the dysregulation of specific miRNAs has been shown to alter insulin sensitivity in target tissues, such as the liver, skeletal muscle, and adipose tissue, thereby contributing to the development and progression of IR [10,11].

Moreover, circulating miRNAs are emerging as potential biomarkers for the early detection of metabolic dysfunction and as therapeutic targets, given their ability to modulate multiple interconnected pathways simultaneously [11,12,13]. In this review, we focus on the mechanistic and therapeutic roles of miRNAs in obesity-induced IR and T2DM, highlighting their regulatory functions in key tissues and potential clinical applications.

## 2. miRNAs: Post-Transcriptional Regulators in Metabolic Biology

miRNAs are a class of endogenous, small non-coding RNAs that function as critical post-transcriptional regulators of gene expression in eukaryotic cells. They are initially transcribed by RNA polymerase II as long primary transcripts (pri-miRNAs), which are sequentially processed in the nucleus by the microprocessor complex, including Drosha and DGCR8, into precursor miRNAs (pre-miRNAs). Pre-miRNAs are exported to the cytoplasm and further cleaved by Dicer into ~22-nucleotide mature miRNA duplexes. One strand of the duplex is then selectively incorporated into the RNA-induced silencing complex (RISC), where it guides the complex to bind partially complementary sequences in target mRNAs [7,13,14].

miRNAs mediate gene silencing predominantly through translational repression and/or target mRNA destabilization and degradation, depending on the level of complementarity and the cellular context. miRNA binding typically occurs within the 3′ untranslated region (3′ UTR) of mRNAs, leading to reduced protein output. A single miRNA can regulate multiple distinct mRNA targets, enabling the coordinated modulation of entire gene networks rather than isolated transcripts [15,16].

In the context of metabolic biology, this regulatory capacity allows miRNAs to regulate critical pathways in insulin signaling, adipogenesis, glucose, and energy homeostasis. miRNAs have been shown to influence key signaling in the insulin receptor pathway, modulate the differentiation and function of adipocytes, and interact with transcription factors that determine metabolic set points in the liver, skeletal muscle, and adipose tissue. Through these mechanisms, miRNAs contribute to the maintenance of metabolic balance and actively participate in the dysregulation observed in obesity, IR, and T2DM [8,9,12].

## 3. miRNAs in Energy Metabolism and Obesity

MicroRNAs have emerged as key regulators of energy metabolism, influencing pathways that determine the balance between energy intake, storage, and expenditure. In obesity, the observed dysregulation of metabolic homeostasis is linked to altered expression profiles of specific miRNAs that modulate processes such as adipogenesis, thermogenesis, lipid metabolism, glucose uptake, and inflammation. Several recent studies demonstrate that miRNAs are involved in the regulation of metabolic fluxes and energy balance at both the cellular and systemic levels, underscoring their relevance to obesity and associated metabolic disorders [15,17,18].

In adipose tissue, miRNAs contribute to the control of white adipocyte differentiation, the browning of white fat depots, and the activation of thermogenesis in brown adipose tissue (BAT). Specific miRNAs have been shown to regulate these processes. For instance, miR-30b/c promotes thermogenic gene expression, including Ucp1 and Cidea, enhancing brown and beige adipocyte function; miR-133a suppresses brown adipogenesis by targeting Prdm16; and miR-27b inhibits browning by repressing key adipogenic and thermogenic factors. These miRNA-mediated effects influence how adipose tissues respond to diverse metabolic demands and environmental stimuli, with important implications for the balance between energy dissipation and storage, central determinants of body weight regulation [19,20,21].

Recent research has highlighted the dynamic role of miRNAs as key mediators linking adipose tissue function with systemic energy homeostasis. Beyond their local regulatory effects, adipose tissue-derived miRNAs may participate in endocrine communication through their incorporation into extracellular vesicles (EVs) [22,23,24,25,26], which are released into circulation and may reach distant metabolic organs [27]. This endocrine-like function supports a potential role for miRNAs in inter-organ communication and coordinated metabolic responses across tissues [27].

**Figure 1:**
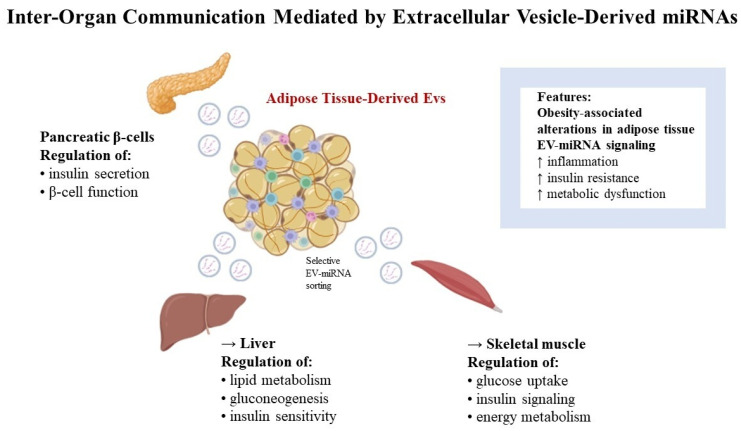
Inter-organ communication mediated by extracellular vesicle-derived miRNAs. Adipose tissue-derived EVs mediate selective miRNA transport to metabolic tissues, including liver, skeletal muscle, and pancreatic β-cells, contributing to the regulation of lipid metabolism, glucose homeostasis, insulin signaling, and β-cell function. In obesity, altered EV-miRNA signaling contributes to inflammation, IR, and metabolic dysfunction. Arrows schematically represent reported associations or regulatory interactions that may be context-, tissue-, or model-dependent. Created in BioRender. Saad, M. (2026). https://BioRender.com/fi024mi (accessed on 23 June 2026).

Moreover, this process is highly regulated and involves sequence-dependent mechanisms that control miRNA sorting and secretion, enabling the selective targeting of recipient tissues. The sequence-dependent sorting of miRNAs into EVs may contribute to inter-organ communication, as demonstrated by Garcia-Martin et al. (2022) [28], who showed that specific miRNA sequence motifs determine EV loading and cellular retention. These findings provide a mechanistic basis for the selective secretion of miRNAs from different cell types and support their potential role in inter-organ communication [28] (Figure 1).

Importantly, altered circulating or EV-associated miRNA levels alone do not establish tissue origin, functional transfer, or target engagement in recipient tissues. Direct evidence of inter-organ miRNA communication requires experimental demonstration of miRNA transfer and functional regulation of target genes in recipient cells or tissues. Although such mechanisms have been demonstrated in selected experimental models, the interpretation of many disease-associated circulating miRNAs remains primarily associative and may be context- or model-dependent.

As mentioned, the regulatory effects of miRNAs extend beyond adipose tissue to key metabolic organs, including the liver and skeletal muscle, where they modulate glucose and lipid metabolism. In the liver, miRNAs such as miR-122, miR-34a, and miR-21 regulate gluconeogenesis, lipid synthesis, and insulin signaling pathways by targeting FOXO1, SIRT1, and SREBP1c, respectively. Dysregulation of these miRNAs has been associated with increased hepatic glucose production and triglyceride accumulation, hallmarks of metabolic dysfunction in obesity and T2DM. These findings were reported in human liver samples and mouse models of diet-induced obesity [29,30,31].

From a functional standpoint, circulating miRNAs can affect insulin signaling cascades in both the liver and skeletal muscle. For example, miR-29a/b impairs insulin receptor substrate 1 (IRS1) expression, miR-143 inhibits AKT activation, and miR-223 modulates phosphoinositide 3-kinase (PI3K) activity, collectively reducing glucose transporter type 4 (GLUT4) translocation and glucose uptake. These effects have been observed in primary human hepatocytes, C2C12 mouse myotubes, and mouse models of obesity [26,27,28]. In the liver, miR-34a and miR-122 also modulate FOXO1 and SREBP1c, promoting gluconeogenesis and lipogenesis, as demonstrated in both murine hepatocytes and human liver biopsies [9,29,32,33,34].

Similarly, in skeletal muscle, miR-1, miR-133a, and miR-206 regulate mitochondrial biogenesis, glucose uptake, and lipid oxidation by targeting PPARGC1A (PGC-1α), IRS1, and MEF2 transcription factors. These regulatory effects, effectively coordinating energy utilization and systemic insulin sensitivity, have been validated in C2C12 mouse myotubes, primary human myoblasts, and mouse models. Taken together, these studies highlight miRNAs as central mediators of inter-organ metabolic homeostasis [35,36,37,38].

In addition to metabolic regulation, miRNAs play a critical role in integrating inflammatory and metabolic signaling. Obesity-associated chronic low-grade inflammation alters miRNA expression profiles in adipose tissue and circulating EVs, leading to the activation of stress-related pathways such as NF-κB and JNK. For example, in high-fat diet (HFD)-induced obese mice, increased expression of miR-155 in adipose tissue macrophages enhances NF-κB signaling by targeting SOCS1, contributing to elevated pro-inflammatory cytokine production and systemic IR [15]. Similarly, miR-146a is upregulated in both mouse and human adipocytes under inflammatory conditions, acting on TRAF6 and IRAK1 to modulate NF-κB activity and cytokine secretion [39,40,41].

These inflammation-responsive miRNAs further exacerbate IR by directly targeting components of the insulin signaling cascade. In HFD rodent models, miR-29a expression was elevated in adipose tissue and associated with the suppression of IRS1 and GLUT4 expression, thereby impairing downstream insulin signaling [17]. In vitro studies using human skeletal muscle cells have further shown that inflammatory stimuli alter miRNA expression profiles, including that of miR-223, contributing to impaired insulin signaling and reduced glucose metabolism under insulin-resistant conditions [9,32,33].

**Figure 2:**
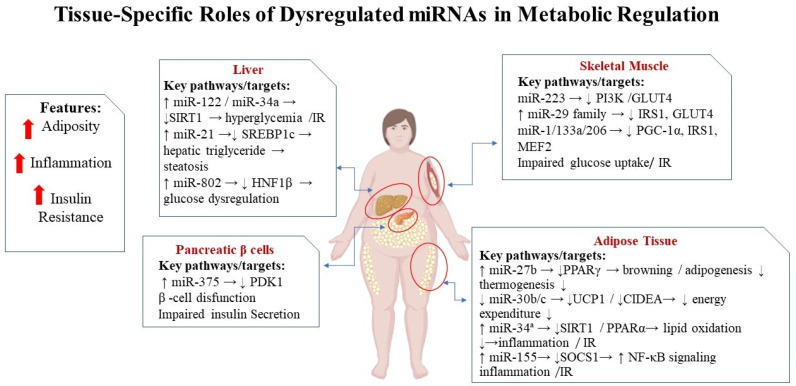
Tissue-specific roles of dysregulated miRNAs in metabolic regulation during obesity, IR, and T2DM. Representative dysregulated miRNAs and their associated pathways in liver, skeletal muscle, adipose tissue, and pancreatic β-cells. Altered miRNA expression contributes to adipose dysfunction, inflammation, impaired glucose uptake, disrupted insulin signaling, and β-cell dysfunction, collectively promoting metabolic dysregulation and IR. Arrows schematically represent reported associations or regulatory interactions that may be context-, tissue-, or model-dependent. Created in BioRender. Saad, M. (2026) https://BioRender.com/fi024mi (accessed on 23 June 2026).

Collectively, these findings provide a strong foundation for investigating how miRNA modulation might restore energy balance and prevent or treat obesity-associated metabolic complications (Figure 2).

## 4. miRNAs and IR

Impaired insulin signaling is a central feature of IR, with IRS1 and IRS2 acting as key mediators of insulin action. miRNA-mediated regulation of these proteins may contribute to defective insulin signaling [42]. For example, miR-144 suppresses IRS1 expression [43], whereas miR-135a directly targets IRS2 and attenuates downstream AKT activation [44]. Experimental inhibition of miR-135a restored IRS2 and phosphorylated AKT levels and improved glucose homeostasis, supporting a mechanistic link between miRNA-mediated IRS regulation and IR [44].

Beyond these mechanistic effects, in obese individuals, changes in circulating miRNA levels are closely linked to IR. For instance, studies by Ortega et al. and Alisi et al. (2014) have highlighted that miR-30d, miR-221, and miR-122 are significantly increased in adolescents with obesity and IR when compared to metabolically healthy adolescents [11,45,46]. Notably, these elevated miRNAs show strong correlations with key clinical indicators such as HOMA-IR, body mass index (BMI), and lipid profiles, implying that they may not only serve as biomarkers but also play a role in the development of metabolic dysfunction [45,46].

Large systematic reviews and human cohort studies have identified multiple circulating miRNAs associated with features of metabolic syndrome, including IR, dyslipidemia, and adiposity across diverse populations. These findings support the utility of employing circulating miRNAs as biomarkers of metabolic impairment in obesity [11,46,47].

miR-143 and miR-103/107 have been implicated in the regulation of adipocyte differentiation. Functional studies in 3T3-L1 mouse pre-adipocytes demonstrated that expression of miR-103 or miR-143 accelerates adipogenesis, leading to increased triglyceride accumulation and the upregulation of adipogenic markers, including PPARγ and FABP4. Their expression is also altered in obesity models, such as leptin-deficient (ob/ob) and diet-induced obese mice [48,49].

Further, miR-27 family members (e.g., miR-27a and miR-27b) act as inhibitors of adipogenesis. In cultured porcine adipocytes and 3T3-L1 cells, overexpression of miR-27a suppresses differentiation and lipid droplet formation, likely by repressing early adipogenic transcription factors such as CREB and interfering with PPARγ induction. In vivo, miR-27 expression is downregulated in mature adipocytes derived from obese mice, suggesting a balancing role in adipose tissue expansion [50,51].

Furthermore, members of the let-7 miRNAs have been implicated in adipogenesis in 3T3-L1 cells, where they regulate growth and differentiation pathways by targeting HMGA2, thereby influencing adipocyte development [52]. Altered expression of the let-7 family has also been reported in adipose tissue isolated from obese individuals, linking these miRNAs to adipose tissue dysfunction and metabolic imbalance [18,52].

Kim et al. (2009) [53] demonstrated that miR-21 regulates adipogenesis and adipose tissue function in human adipose-derived stem cells (hASCs) and mouse mesenchymal models. Its actions involve the modulation of TGF-β signaling and other pathways relevant to lipid storage and differentiation [53].

Additional studies have implicated miRNAs, including miR-34a and miR-155, as modulators of adipose tissue inflammation and systemic insulin sensitivity. In HFD-fed mice and human adipocytes, miR-34a was shown to regulate SIRT1 and PPARα, thus linking adipogenesis to energy metabolism, while miR-155 modulates SOCS1 and NF-κB signaling, thereby establishing a connection between inflammation and IR [39,54].

It was demonstrated that a reduction in caveolin-1 expression within liver and adipose tissue through miR-103/107 impairs insulin signaling. This effect was demonstrated in HFD-fed mice as well as in primary human hepatocytes, thus highlighting a conserved mechanism contributing to IR in obesity [49].

Hepatic glucose dysregulation is also influenced by elevated miR-802, which targets hepatocyte nuclear factor 1β (HNF1β) and modulates genes involved in glucose metabolism. Kornfeld et al. (2013) demonstrated, using HFD-induced obese mice and human liver samples, that the obesity-associated overexpression of miR-802 impairs glucose metabolism and contributes to systemic IR via suppression of HNF1β signaling [55].

In skeletal muscle and liver, members of the miR-29 family (including miR-29a/b/c) have been implicated in impaired insulin signaling through modulation of insulin-responsive pathways, including IRS1 and glucose transport mechanisms. Functional studies in skeletal muscle cells and obesity-related models indicate that increased miR-29 expression is associated with reduced glucose uptake and diminished insulin responsiveness, thereby supporting a pathogenic role in metabolic dysfunction [56].

Finally, Poy et al. (2004) [57] showed that the regulation of insulin secretion in pancreatic β-cells is controlled by miR-375, which targets PDK1 and other components of the exocytotic machinery. Functional studies in MIN6 β-cell lines and murine pancreatic islets have shown that altered miR-375 expression impairs glucose-stimulated insulin secretion, further supporting a role for this miRNA in β-cell dysfunction associated with T2DM [57].

Experimental modulation of these miRNAs could restore insulin sensitivity, highlighting their potential as therapeutic targets. Comparative studies have revealed tissue-specific and context-dependent effects, underscoring the need for precise interventions.

To facilitate comparison of the representative miRNAs discussed throughout this review, Table 1 summarizes their primary tissue or biofluid, validated targets or biological pathways, type of supporting evidence, and potential clinical application.

Building on these findings, the next section explores circulating and tissue-specific miRNA profiles in T2DM, gleaning insights into their diagnostic, prognostic, and mechanistic relevance.

## 5. miRNA Profiles in T2DM

In T2DM, several circulating miRNAs are found to be consistently dysregulated and are associated with key pathological features of the disease, including endothelial dysfunction, impaired insulin secretion, and overall metabolic dysregulation. For example, reduced levels of miR-126 have been widely reported in the plasma of individuals presenting with T2DM relative to healthy controls, and lower circulating miR-126 levels correlate with markers of endothelial dysfunction and diabetic complications in clinical cohorts [13,58].

Circulating miR-21 levels have also been identified as being altered in T2DM, with higher plasma levels observed in subjects with impaired glucose metabolism and elevated inflammatory markers. These increases in miR-21 are correlated with indices of glycemic control, such as fasting glucose and HbA1c, suggesting that miR-21 may reflect systemic inflammation and metabolic stress associated with T2DM [59].

miR-375, which is abundant in pancreatic islets, is elevated in the blood of people with T2DM and in their close relatives who are at high risk. This increase is linked to β-cell dysfunction and reduced insulin secretion, supporting miR-375 as a potential biomarker of early β-cell stress and diabetes risk [13,60].

Beyond individual miRNAs, integrated profiles of circulating miRNAs have demonstrated improved discrimination between T2DM patients and controls. For example, multi-miRNA signatures, including miR-30 family members and other miRNAs, have been identified and correlate with IR and disease progression, indicating that combined miRNA panels may enhance the diagnostic and prognostic utility of miRNA profiling in T2DM [61,62].

Moreover, clinical evidence shows that specific miRNAs are consistently dysregulated in the circulation and are associated with disease mechanisms, clinical status, and complications. Several cohort profiling studies have identified patterns of altered circulating miRNA expression that differentiate T2DM patients from healthy controls, prediabetic individuals, and subjects with varying degrees of IR or β-cell dysfunction [46,61,63].

Functional analysis of serum miR-375, which is enriched in pancreatic islets, shows that this miRNA is elevated in patients with T2DM and first-degree relatives at increased risk, correlating with impaired glucose-stimulated insulin secretion. This suggests that miR-375 reflects β-cell stress and dysfunction before the onset of overt hyperglycemia, making it a promising indicator of early disease susceptibility [64].

Other miRNAs, including miR-34a and miR-146a, have also been implicated in the pathophysiology of T2DM. Elevated miR-34a levels have been reported in overweight and obese subjects with T2DM, whereas miR-146a levels may be reduced in patients with diabetic complications such as nephropathy, indicating that these miRNAs reflect metabolic stress and disease severity [65,66].

Multi-miRNA profiling studies have identified circulating panels that include miR-30a-5p, miR-122, miR-29 family members, and miR-375, which show differential expression in T2DM as compared to controls. These multi-marker signatures correlate with IR, glycemic control, and disease progression more strongly than individual miRNAs, supporting their utility in diagnostic and prognostic applications [43,67].

Overall, the dysregulation of these circulating miRNAs in T2DM supports their potential role as biomarkers of metabolic and inflammatory dysfunction. However, associations between circulating miRNA profiles and tissue-specific disease mechanisms should be interpreted cautiously unless tissue origin and functional target engagement have been directly demonstrated.

## 6. Therapeutic and Translational Implications of miRNAs

Although substantial progress has been made in understanding the mechanistic roles of miRNAs in obesity, IR, and T2DM, an increasing body of evidence supports their broader relevance beyond pathophysiological regulation. Due to their ability to modulate multiple interconnected signaling pathways and mediate inter-organ communication, miRNAs have emerged as promising candidates for clinical translation. In this context, growing efforts have focused on exploring miRNAs as therapeutic targets, predictive biomarkers, and modulators of treatment response. This section discusses current therapeutic strategies, translational advances, and clinical evidence supporting the application of miRNAs in obesity-associated metabolic disorders.

### 6.1. Therapeutic Strategies and Future Perspectives

Unlike conventional therapeutic approaches that often focus on single molecular targets, miRNAs can simultaneously regulate metabolic and inflammatory pathways involved in the progression of obesity, IR, and T2DM. In this context, specific miRNAs such as miR-30c, miR-223, and miR-34a have demonstrated mechanistic roles in controlling lipid metabolism, glucose uptake, and insulin signaling in preclinical models, making them attractive candidates for therapeutic intervention [54,68].

miR-30c is downregulated in individuals with T2DM and contributes to dyslipidemia and impaired hepatic insulin signaling. Functional studies in HFD-fed mice and human hepatocyte cultures indicate that restoring miR-30c improves lipid metabolism, reduces hepatic triglyceride accumulation, and enhances insulin responsiveness, highlighting its therapeutic potential [54,68,69].

miR-223 has also emerged as a regulator of glucose metabolism and insulin signaling in adipose tissue and skeletal muscle. Altered expression of miR-223 has been associated with IR and impaired glucose homeostasis in obesity and T2DM. Experimental modulation of miR-223 in preclinical models suggests a role in metabolic regulation, although the underlying mechanisms and tissue-specific effects remain incompletely understood [33,69].

Finally, miR-34a is upregulated in both obese and diabetic adipose tissue and contributes to IR and chronic inflammation by targeting SIRT1 and PPARα. Knockdown experiments in mice improve adipocyte function, reduce inflammatory signaling, and enhance systemic glucose tolerance, indicating that miR-34a antagonism could be a viable strategy for restoring metabolic control [54].

Despite growing preclinical and translational interest, no miRNA-based therapeutic has yet received regulatory approval for the treatment of obesity, IR, or T2DM, and miRNA mimics and anti-miRs remain investigational strategies [70]. Although other RNA-based therapeutics, such as the siRNA inclisiran [71], have entered clinical use, these approaches are mechanistically distinct from miRNA-targeted therapies [72].

In parallel, emerging artificial intelligence-assisted approaches may further support the design and optimization of small nucleic acid therapeutics by improving sequence selection, predicting off-target interactions, and assisting the evaluation of pharmacological and safety-related properties [73].

Investigational miRNA-based interventions include anti-miRs, miRNA mimetics, and EV-mediated delivery, which permit the simultaneous modulation of multiple metabolic pathways. Anti-miRs are chemically modified oligonucleotides that inhibit specific miRNAs. For instance, administration of anti-miR-34a in HFD-fed mice reduces adipose tissue inflammation, improves systemic glucose tolerance, and enhances insulin signaling. Similarly, anti-miR-155 decreases NF-κB-mediated pro-inflammatory cytokine production in adipose tissue macrophages, thereby mitigating IR. These studies demonstrate that the targeted inhibition of overexpressed miRNAs can restore metabolic balance in obesity and IR [54,74,75].

**Figure 3:**
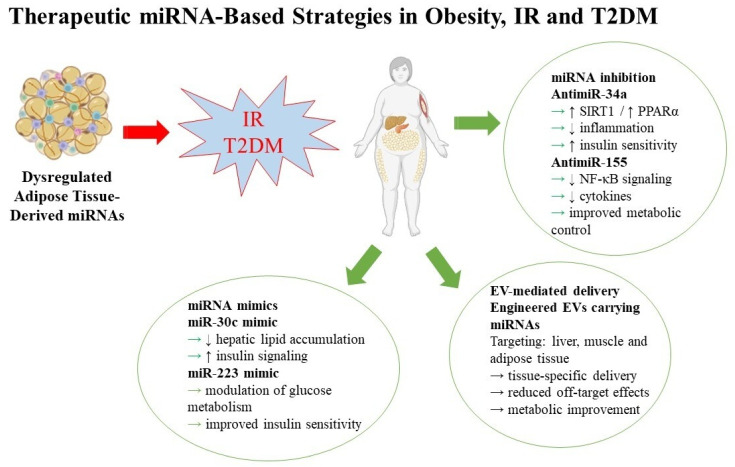
Therapeutic strategies targeting miRNAs in obesity, IR, and T2DM. Dysregulated miRNAs contribute to metabolic dysfunction and represent promising therapeutic targets in obesity-associated IR and T2DM. Current miRNA-based interventions include miRNA inhibition using anti-miRs (e.g., anti-miR-34a and anti-miR-155), the restoration of beneficial miRNAs through miRNA mimics (e.g., miR-30c and miR-223 mimics), and EV-mediated delivery systems designed to improve tissue-specific targeting and reduce off-target effects. These approaches aim to improve insulin sensitivity, reduce inflammation, and restore metabolic homeostasis. Arrows schematically represent reported associations or regulatory interactions that may be context-, tissue-, or model-dependent. Created in BioRender. Saad, M. (2026) https://BioRender.com/fi024mi (accessed on 23 June 2026).

miRNA mimetics are synthetic molecules designed to restore the function of downregulated miRNAs. In murine models, delivery of miR-30c mimetics to hepatocytes improves lipid metabolism, reduces hepatic triglyceride accumulation, and enhances insulin sensitivity [68]. These approaches exemplify how supplementing deficient miRNA can ameliorate specific pathogenic pathways (Figure 3).

Engineered EVs and lipid nanoparticle (LNP)-based delivery systems have demonstrated improved cargo stability, enhanced tissue specificity, and reduced off-target effects in preclinical studies [27,28]. These technologies enable more efficient delivery of therapeutic miRNAs to metabolically relevant tissues, including adipose tissue, liver, skeletal muscle, and pancreatic islets [76,77,78,79].

For example, adipose tissue-derived EVs carrying miR-126 or miR-21 modulate endothelial function and glucose homeostasis within the liver and skeletal muscle of murine models. Sequence-directed sorting further enables selective loading of therapeutic miRNAs, increasing efficacy while minimizing off-target effects [58,80] (Figure 3).

Together, these technological advances represent important steps toward overcoming current barriers related to delivery efficiency, tissue targeting, and therapeutic safety, facilitating the clinical translation of miRNA-based therapies for the treatment of metabolic disorders.

Despite these promising strategies, several challenges remain, specifically achieving efficient and tissue-specific delivery, avoiding immune clearance, controlling dosage and stability, and ensuring long-term therapeutic effects. Ongoing preclinical studies aim to optimize these parameters and identify the most effective miRNA candidates for metabolic disorders, laying the groundwork for subsequent clinical and translational research.

### 6.2. Clinical and Translational Evidence

**Figure 4:**
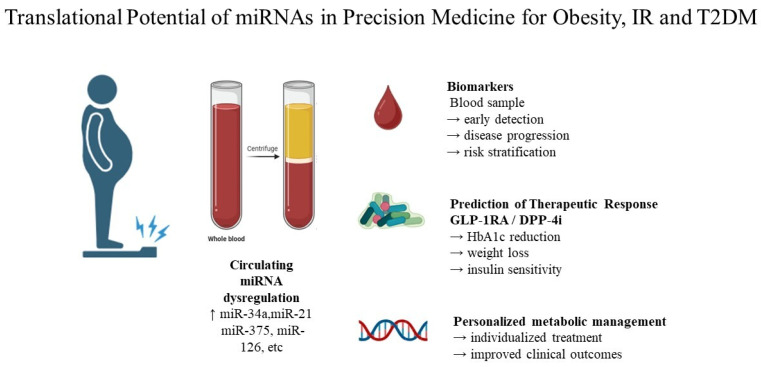
Translational potential of circulating miRNAs in precision medicine for obesity, IR, and T2DM. Circulating miRNA signatures provide clinically relevant information for metabolic disease management, supporting early diagnosis, disease stratification, and therapeutic monitoring in obesity-associated metabolic disorders. In addition, specific miRNA profiles may predict responsiveness to treatments such as GLP-1 receptor agonists (GLP-1 RAs) and DPP-4 inhibitors, enabling individualized therapeutic strategies and improved metabolic outcomes. The translational applications shown remain investigational and do not represent established clinical practice. Created in BioRender. Saad, M. (2026) https://BioRender.com/fi024mi (accessed on 23 June 2026).

Our expanding understanding of miRNA-mediated regulatory networks in metabolic tissues has emboldened efforts to translate these findings into clinical applications. In humans, circulating and tissue-derived miRNAs have emerged as promising tools for disease stratification, risk prediction, and therapeutic monitoring in metabolic disease [81,82,83]. Their stability in biofluids, accessibility through minimally invasive sampling, and ability to reflect dynamic metabolic changes position them as attractive candidates for clinical use [83] (Figure 4).

#### 6.2.1. Circulating miRNAs as Biomarkers

Circulating miRNAs have been consistently identified as biomarkers of metabolic dysfunction across human populations. Several miRNAs, including miR-27, miR-30a, miR-34a, miR-93, miR-146a, and miR-192, are differentially expressed in individuals with obesity, pre-diabetes, and T2DM and are closely associated with IR, chronic low-grade inflammation, and β-cell dysfunction. These associations have been demonstrated in cross-sectional and longitudinal studies, where circulating miRNA levels correlate with key clinical parameters such as HOMA-IR, fasting glucose levels, HbA1c, lipid profiles, and inflammatory markers [13,16,83,84].

Mechanistically, many of these miRNAs are linked to specific pathogenic pathways. For example, miR-122 is associated with hepatic lipid metabolism and steatosis; miR-126 reflects endothelial function and vascular integrity, while miR-34a is linked to inflammation and mitochondrial dysfunction. Alterations in their circulating levels may reflect disease-associated metabolic changes; however, circulating abundance alone does not establish tissue origin or direct functional involvement in the underlying pathogenic pathways [29,54,67].

More recently, extracellular vesicle-associated miRNAs (EV-miRNAs) have emerged as particularly promising biomarkers in obesity-related metabolic disorders. Unlike freely circulating miRNAs, EV-miRNAs are protected from enzymatic degradation and may more accurately reflect the physiological (or pathophysiological) state of their tissue of origin. Moreover, adipose tissue is considered a major source of circulating EV-miRNAs in obesity, and specific adipose-derived EV-miRNA signatures have been associated with the development of IR, chronic inflammation, dyslipidemia, and T2DM [22,77].

Beyond individual miRNAs, integrated circulating miRNA signatures have demonstrated superior diagnostic and prognostic performance. miRNA panels have revealed an ability to predict disease progression, metabolic deterioration, and responsiveness to interventions, highlighting the advantage of multi-marker approaches in capturing metabolic complexity [83,85].

Collectively, these findings support the potential clinical utility of circulating and EV-associated miRNAs as minimally invasive biomarkers for early diagnosis, risk stratification, and therapeutic monitoring, reinforcing their relevance to precision medicine approaches in obesity-associated IR and T2DM.

#### 6.2.2. miRNAs as Predictors of the Therapeutic Response

Circulating miRNAs have also emerged as potential predictive biomarkers of the therapeutic response in metabolic diseases. Evidence from clinical and translational studies indicates that baseline levels of specific miRNAs may predict improvements in glycemic control, body weight reduction, and cardiometabolic outcomes following treatment with GLP-1RAs [86].

In particular, circulating miR-15a-5p has been associated with greater weight loss, whereas elevated miR-126-3p and miR-378-3p levels correlate with improved HbA1c reduction and metabolic responses after therapy. Moreover, GLP-1RAs modulate several miRNAs involved in endothelial function, inflammation, adipogenesis, and glucose metabolism, supporting the concept that miRNA signatures may contribute to patient stratification, thereby justifying precision-based medical approaches in obesity and T2DM [85,87,88,89,90].

Similarly, treatment with dipeptidyl peptidase-4 (DPP-4) inhibitors and insulin therapy alters circulating miRNA expression patterns. These changes often parallel improvements in metabolic control and may distinguish responders from non-responders in longitudinal studies, reinforcing the role of miRNAs as dynamic indicators of the therapeutic response [91].

Together, these findings indicate that miRNA profiling may not only serve as a biomarker of disease progression but also facilitate the identification of individuals most likely to benefit from specific therapeutic interventions. However, current clinical evidence is derived predominantly from exploratory, observational, and cohort studies, whereas independent validation cohorts and registered clinical studies remain limited. Consequently, although several circulating miRNAs show considerable promise as biomarkers and predictors of therapeutic response, further validation is required before routine clinical implementation.

#### 6.2.3. Registered Clinical Studies

Registered and ongoing clinical studies investigating miRNAs in metabolic disorders primarily focus on their potential as biomarkers and on disease- or treatment-associated miRNA profiles. For example, clinical trial NCT02459106 is investigating adipose tissue-released miRNAs in relation to skeletal muscle biology and insulin sensitivity in humans, aiming to characterize associations between adipose tissue-derived miRNA profiles and systemic metabolic outcomes [92].

In addition, multiple observational and cohort studies have explored circulating miRNA profiles in relation to glycemic control, IR, and response to pharmacological therapies, supporting their potential integration into clinical workflows for diagnosis and monitoring [46,83,93,94,95]. These studies provide clinical and biomarker-oriented evidence but should be distinguished from therapeutic trials directly evaluating miRNA-based interventions, which remain investigational.

Despite these advances, several challenges remain. Variability in miRNA quantification methods, the lack of standardized normalization strategies, and heterogeneity across study populations limit reproducibility. Differences in sample type, pre-analytical conditions, and detection platforms further complicate comparisons between studies. Addressing these limitations will be essential for establishing reliable and clinically applicable miRNA-based assays [96].

Overall, continued integration of mechanistic insights with well-designed clinical studies will be critical to fully realize the potential of miRNAs in precision medicine. As methodological standardization improves and larger cohorts are analyzed, circulating miRNAs may play an increasingly important role in the diagnosis, prognosis, and individualized treatment of T2DM and associated metabolic disorders.

## 7. Conclusions

miRNAs are important regulators of metabolic homeostasis, influencing key processes such as adipogenesis, insulin signaling, inflammation, and energy balance across multiple tissues. Their dysregulation is associated with obesity, IR, and T2DM, with altered miRNA profiles reported in adipose tissue, liver, skeletal muscle, and pancreatic islets.

Circulating miRNAs have emerged as potential non-invasive biomarkers, reflecting systemic metabolic alterations and correlating with clinical parameters such as IR and glycemic control. In addition, specific miRNA signatures show potential in predicting therapeutic response, although their application in precision medicine requires further clinical validation and standardization.

miRNA-based therapeutic strategies have shown promising effects in preclinical models; however, challenges related to delivery, specificity, and long-term safety still limit their clinical use. Future research in well-characterized human populations and the development of standardized methodological approaches will be essential to define the clinical utility of miRNAs as biomarkers and therapeutic targets in metabolic disorders.

## Figures and Tables

**Table 1:** Key miRNAs associated with obesity, insulin resistance (IR), and type 2 diabetes mellitus (T2DM).

miRNA	Tissue/Biofluid	Disease Context	Validated Target(s)/Pathway	Type of Evidence	Translational Relevance
miR-30b/c	Brown/beige adipose tissue	Obesity	UCP1, CIDEA	Mechanistic	Thermogenesis; therapeutic candidate
miR-133a	Brown adipose tissue; skeletal muscle	Obesity	PRDM16	Mechanistic	Regulator of brown adipogenesis
miR-27a/b	White adipose tissue	Obesity	PPARγ	Mechanistic	Regulator of adipogenesis
miR-122	Liver; plasma	Obesity, T2DM	FOXO1, SREBP1c	Mechanistic + Biomarker	Biomarker; therapeutic candidate
miR-34a	Liver; adipose tissue	Obesity, IR	SIRT1, PPARα	Mechanistic + Therapeutic	Anti-miR therapeutic candidate
miR-21	Liver; circulation	Obesity, T2DM	SREBP1c	Mechanistic + Biomarker	Biomarker; therapeutic candidate
miR-155	Adipose tissue macrophages	Obesity-associated inflammation	SOCS1, NF-κB	Mechanistic + Therapeutic	Anti-miR strategy
miR-146a	Adipose tissue; circulation	Obesity, T2DM	IRAK1, TRAF6	Mechanistic + Biomarker	Biomarker; inflammatory regulator
miR-29 family	Liver; skeletal muscle	Insulin resistance	IRS1, GLUT4	Mechanistic	Therapeutic target
miR-143	Skeletal muscle	Insulin resistance	AKT signaling	Mechanistic	Therapeutic target
miR-223	Adipose tissue; skeletal muscle	Insulin resistance	PI3K signaling	Mechanistic	Therapeutic candidate
miR-144	Skeletal muscle	Insulin resistance	IRS1	Mechanistic	Regulator of insulin signaling
miR-135a	Liver	Insulin resistance	IRS2, AKT	Mechanistic	Regulator of insulin signaling
miR-103/107	Liver; adipose tissue	Obesity, IR	Caveolin-1	Mechanistic	Therapeutic candidate
miR-802	Liver	Obesity, IR	HNF1β	Mechanistic	Regulator of glucose metabolism
miR-375	Pancreatic β-cells; serum	T2DM	PDK1	Mechanistic + Biomarker	Biomarker of β-cell dysfunction
miR-126	Plasma	T2DM	Endothelial pathways	Biomarker	Diagnostic biomarker
miR-15a-5p	Circulation	T2DM	Treatment-associated	Clinical biomarker	Predictor of GLP-1RA response
miR-126-3p/miR-378-3p	Circulation	T2DM	Treatment-associated	Clinical biomarker	Predictors of therapeutic response

## Data Availability

No new data were created or analyzed in this study. Data sharing is not applicable to this article.
